# Supplementary material for: Causal interplay between lactose intolerance and gut microbiota: a combined bidirectional Mendelian randomization and in vivo validation study
Source: Front Nutr. 2026 Jun 1;13:1803337. doi: 10.3389/fnut.2026.1803337 (PMC13265576; doi:10.3389/fnut.2026.1803337)
Supplement: Supplementary file 1 [file Data_Sheet_1.zip › supplementary materials/Forward/leave one out cross validation/ebi-a-GCST90027622.finngen_R12_E4_LACTONAS.pdf]

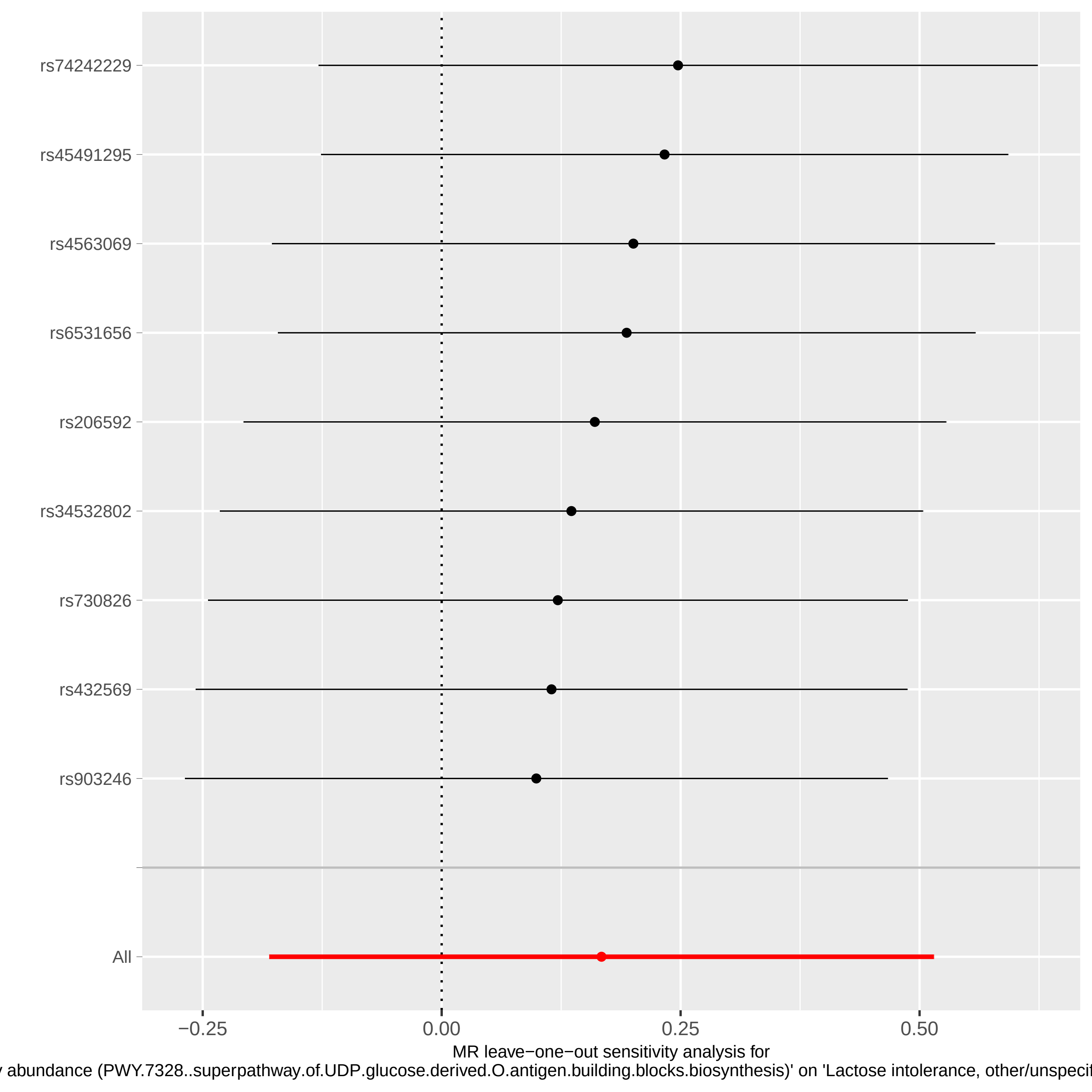

rs74242229
rs45491295
rs4563069
rs6531656
rs206592
rs34532802
rs730826
rs432569
rs903246
All
−0.25
0.00
0.25
0.50
MR leave−one−out sensitivity analysis for
abundance (PWY.7328..superpathway.of.UDP.glucose.derived.O.antigen.building.blocks.biosynthesis)' on 'Lactose intolerance, other/unspecif
